# Vacancy-induced dislocations within grains for high-performance PbSe thermoelectrics

**DOI:** 10.1038/ncomms13828

**Published:** 2017-01-04

**Authors:** Zhiwei Chen, Binghui Ge, Wen Li, Siqi Lin, Jiawen Shen, Yunjie Chang, Riley Hanus, G. Jeffrey Snyder, Yanzhong Pei

**Affiliations:** 1 Key Laboratory of Advanced Civil Engineering Materials of Ministry of Education, School of Materials Science and Engineering, Tongji University, 4800 Caoan Road, Shanghai 201804, China; 2 Beijing National Laboratory for Condensed Matter Physics, Institute of Physics, Chinese Academy of Science, Beijing 100190, China; 3 Department of Materials Science and Engineering, Northwestern University, 2220 Campus Drive, 3033 Cook Hall, Evanston, Illinois 60208 USA

## Abstract

To minimize the lattice thermal conductivity in thermoelectrics, strategies typically focus on the scattering of low-frequency phonons by interfaces and high-frequency phonons by point defects. In addition, scattering of mid-frequency phonons by dense dislocations, localized at the grain boundaries, has been shown to reduce the lattice thermal conductivity and improve the thermoelectric performance. Here we propose a vacancy engineering strategy to create dense dislocations in the grains. In Pb_1−*x*_Sb_2*x*/3_Se solid solutions, cation vacancies are intentionally introduced, where after thermal annealing the vacancies can annihilate through a number of mechanisms creating the desired dislocations homogeneously distributed within the grains. This leads to a lattice thermal conductivity as low as 0.4 Wm^−1^ K^−1^ and a high thermoelectric figure of merit, which can be explained by a dislocation scattering model. The vacancy engineering strategy used here should be equally applicable for solid solution thermoelectrics and provides a strategy for improving *zT*.

## Abstract (web summary)

In thermoelectric materials, dislocations at grain boundaries can be used to scatter midfrequency phonons. Here, Chen *et al*. use vacancy engineering and thermal annealing to generate dislocations homogeneously within the crystalline grains and obtain good figures of merit for PbSe-based thermoelectrics.

Thermoelectric materials attract increasing interest, driven by the world-wide demand for clean energy^1^. Based on either the Seebeck or Peltier effects, thermoelectrics can be used for power generation or for refrigeration, respectively. Thermoelectrics use the charge carriers inside a material as a working medium, therefore enabling a vibration-free and emission-free solution to the direct conversion between heat and electricity. The biggest challenge is to enhance the conversion efficiency, which is mostly limited by the materials' dimensionless figure of merit, *zT*=*S*^2^*T*/*ρ*(*κ*_E_+*κ*_L_), where *S* is the Seebeck coefficient, *ρ* is the electric resistivity, *κ*_E_ and *κ*_L_ are the electronic and the lattice contribution to the thermal conductivity, and *T* is the absolute temperature.

Owing to the strong coupling between *S*, *ρ* and *κ*_E_, two straightforward strategies for improving *zT* are to increase the electrical performance via the power factor *S*^2^/*ρ* and to reduce the independent material property, lattice thermal conductivity *κ*_L_. The former strategy has been recently demonstrated by the band engineering concept^2^,^3^,^4^,^5^,^6^,^7^,^8^,^9^,^10^ and the later one can be accomplished by a few approaches that include nanostructuring^11^,^12^,^13^,^14^, alloying^15^,^16^ and lattice anharmonicity^17^,^18^.

Among all the demonstrated approaches for achieving a low lattice thermal conductivity mentioned above, the essential commonality is the strengthened phonon scattering, but in different ways. Specifically, nanostructuring increases boundary scattering and alloying introduces point defect scattering, whereas anharmonic lattice vibrations lead to a strong inherent phonon–phonon scattering. These different scattering mechanisms collectively contribute to shorten the overall relaxation time (*τ*_tot_) of phonons, each with a characteristic frequency (*ω*) dependence, leading to a low lattice thermal conductivity. More quantitatively, boundary scattering by nanostructures leads to a relaxation time, *τ*_B_∝*ω*^0^, which enables effective scattering of low-frequency phonons; point defect scattering in alloys results in a relaxation time, *τ*_PD_∝*ω*^−4^, effectively scattering the high-frequency phonons, and the inherent phonon–phonon scattering by anharmonic lattice vibrations has respectively a relaxation time, *τ*_U,N_∝*ω*^−2^, for Umklapp (*τ*_U_) and normal (*τ*_N_) processes, which enables effective scattering on phonons with all frequencies.

Although it is rarely focused on, dislocation scattering is another process that can be effective at reducing lattice thermal conductivity in thermoelectrics^19^,^20^. The frequency dependence of phonon scattering on dislocation strian fields (*τ*_DS_∝*ω*^−1^) and dislocation cores (*τ*_DC_∝*ω*^−3^) makes this mechanism particularly effective at scattering mid-range frequency phonons, phonons missed by the previously discussed scattering mechanisms^21^,^22^. Even with scattering by grain boundary dislocations, which are known to have much shorter range strain fields than lattice dislocations^23^, a large *κ*_L_ reduction has been recently observed experimentally^20^. Therefore, it is believed that phonon scattering by dislocations, whether in the grain boundary structure or in the lattice, is an effective approach for advancing thermoelectrics.

Dislocations are generally undesired in semiconductors and ceramics^24^,^25^ when the thermal conductivity is not a major functionality, due to their detrimental effects on the mechanical properties, carrier mobility and optical properties^26^,^27^,^28^. To achieve an effective *κ*_L_ reduction by dislocations in thermoelectric semiconductors, a simple estimation shows that the density of uniformly distributed dislocations within the grains (*N*_D_) needs to be approaching 10^12^ cm^−2^ or higher^29^, being at least two orders of magnitude higher than that can be normally obtained in semiconductors^30^. The most straightforward way to introduce dislocations into a material is through plastic deformation. However, most thermoelectrics do not readily plastically deform and dislocations must be introduced by other means.

In thermoelectrics, dislocations have been introduced through the precipitation of secondary phases where misfit dislocations mediate the lattice mismatch at coherent phase boundaries^16^. A large density of grain boundary dislocations has also been achieved by liquid-phase compaction (*N*_D_=2 × 10^11^ cm^−2^)^20^. Another method for introducing a large density of dislocations is through the introduction of a large concentration of vacancies^31^. On thermal annealing these vacancies will diffuse forming vacancy clusters of lower energy, these vacancy clusters then collapse into closed loops of edge dislocations. This mechanism was initially observed in metals^32^ and has since been observed in ionic crystals^33^. In addition, vacancies in a material facilitate dislocation climb and at vacancy concentrations far from equilibrium the resulting dislocation motion can increase the dislocation content (that is, the activation of Bardeen–Herring sources)^34^,^35^.

Frank^34^ highlights a situation where the concentration of point defects is far from equilibrium, which is common in semiconducting materials^31^. When annealed, the resulting dislocation motion via climb will result in an increase in dislocation content^34^. Although these mechanisms are well understood for ionic crystals where vacancies are produced through Frenkel defects or Schottky pairs, they are likely to be more complicated in systems where vacancies are introduced via charge compensation doping and one vacancy-type dominates. These mechanisms lead to a strategy for obtaining uniformly distributed dense dislocations through vacancy engineering in thermoelectric materials.

Compared with the solvent compound, when a solute of a smaller cation-to-anion ratio is dissolved forming a solid solution, either vacancies at the cation site or interstitial anions would be expected. Anion interstitials generally have a much larger defect formation energy than that of a cation site vacancy, because typically the anion radius is larger than that of the cation. This leads to the preferred formation of cation vacancies. Thus, the formation of dislocations would be expected due to the collapse of cation vacancy clusters. It is expected that the density of the dislocations can be easily controlled by tuning the composition of the solid solution.

This work focuses on the thermoelectric properties of PbSe-Sb_2_Se_3_ solid solutions (Pb_1−*x*_Sb_2*x*/3_Se) to demonstrate this strategy. To maintain a charge-balanced, undoped semiconductor within the solid solution, with rock salt structure, it is believed that for three Pb sites replaced by two Sb atoms and a third site becomes vacant. The resulting cation vacancies lead to a high density of uniformly distributed dislocations (Fig. 1a,b). These dislocations provide a strong scattering of phonons in the mid-range of frequencies (*τ*_DC_∝*ω*^−3^, *τ*_DS_∝*ω*^−1^). This is in addition to the point defect scattering (*τ*_PD_∝*ω*^−4^) from the Sb/Pb substitution for high-frequency phonons and strong inherent phonon–phonon scattering (*τ*_U,N_∝*ω*^−2^) for phonons at all frequencies. This full frequency spectrum scattering of phonons, with an emphasis on the mid-frequency phonons, leads to the low value lattice thermal conductivity (*κ*_L_), compared with intrinsic PbSe^36^, approaching the amorphous limit estimated by assuming a phonon mean free path of minimal distance^37^ in a broad temperature range (Fig. 1c). Most importantly, the ultralow lattice thermal conductivity of ∼0.4 Wm^−1^ K^−1^ leads to higher *zT* than previously reported in PbSe (Fig. 1d). This vacancy engineering strategy for dislocation scattering to reduce the lattice thermal conductivity should be widely applicable in thermoelectric solid solutions.

## Results

### Microstructure and formation mechanisms of dislocations

The powder X-ray diffraction (XRD) indicates all the materials obtained here crystalize in NaCl structure with high phase purity (Supplementary Fig. 1a). The formation of Pb_1−*x*_Sb_2*x*/3_Se solid solution in this work can be evident from the linear decrease in lattice parameter with increasing *x* (Supplementary Fig. 1b), which agrees well with the Vegard's law. Neither precipitates nor nanometre-scale grains are observed according to our XRD analyses and transmission electron microscope (TEM) observations.

As the cation-to-anion ratio in Sb_2_Se_3_ is 1/3 smaller than that in PbSe, formation of a rock-salt structured Pb_1−*x*_Sb_2*x*/3_Se solid solution would lead to *x*/3 negatively charged vacancies at the Pb site per formula unit rather than interstitial Se. Pb deficiency can be observed in the energy dispersive X-ray spectroscopy analysis. These Pb vacancies can increase the dislocation content through a number of mechanisms that have been observed in ionic crystals^33^,^23^,^38^,^39^. One possibility is the activation of Bardeen–Herring sources (the climb analogue to the Frank–Read source) where vacancy diffusion facilitates dislocation climb^34^. These sources act as dislocation multiplication sites and are diffusion-limited processes. In addition, dislocations in ionic crystals can have an electrostatic charge^23^,^38^. These charged regions interact with the negatively charged Pb vacancies retarding the motion of the dislocations via glide stopping their annihilation with each other or at a surface.

**Figure 1:**
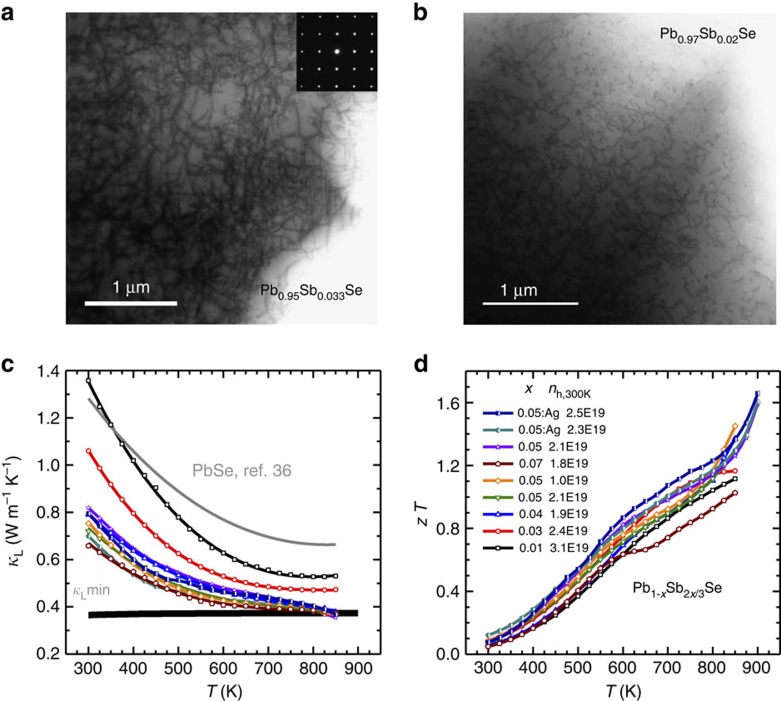
Microstructures and thermoelectric properties. Uniformly distributed dense dislocations in Pb_0.95_Sb_0.033_Se solid solution (**a**) and Pb_0.97_Sb_0.02_Se solid solution (**b**). The temperature-dependent lattice thermal conductivity (**c**) and thermoelectric figure of merit (**d**) for Pb_1−*x*_Sb_2*x*/3_Se (*x*=0.01, 0.03, 0.04, 0.05 and 0.07) with or without Ag doping. The lattice thermal conductivity of PbSe^36^ (grey line) and estimated minimal lattice thermal conductivity (black line) are included for comparison. Dislocations due to cation vacancies lead to the lowest lattice thermal conductivity and the highest *zT* in PbSe thermoelectrics.

The samples in this study were annealed at 1,023 K for 2 days, giving the system sufficient thermal energy to activate the above mentioned, diffusion-limited dislocation nucleation and multiplication processes. These dislocations can subsequently climb, glide and tangle into the complex dislocation network seen in Fig. 1a,b. Although these mechanisms are well understood for ionic crystals where vacancies are produced through Frenkel defects or Schottky pairs, they are likely to be more complicated in systems where vacancies are introduced via charge compensation doping and one vacancy type dominates. Different thermal histories, composition and synthesis methods may explain why other Pb_1−*x*_Sb_*x*_Se studies show the formation of Sb-rich impurity phases but not dislocations^40^.

According to the theory of impurity diffusion, in particular in cubic crystals with vacancies^41^,^42^,^43^, the existence of vacancies will significantly retard the diffusion of the substitutional impurities because of the coulombic interaction between them^34^,^36^,^44^,^45^, whereas the diffusion of vacancies themselves can be largely accelerated when they are bound with dislocations^46^. Therefore, the substitutional Sb impurity on the Pb sites do not diffuse fast enough to form an aggregation of Sb-rich phases as confirmed by the TEM observations for Pb_1−*x*_Sb_2*x*/3_Se. This mechanism may further explain the formation of Sb-rich impurity phases but not dislocations in Pb_1−*x*_Sb_*x*_Se, which may have fewer cation vacancies^40^.

### Density of dislocation

**Figure 2:**
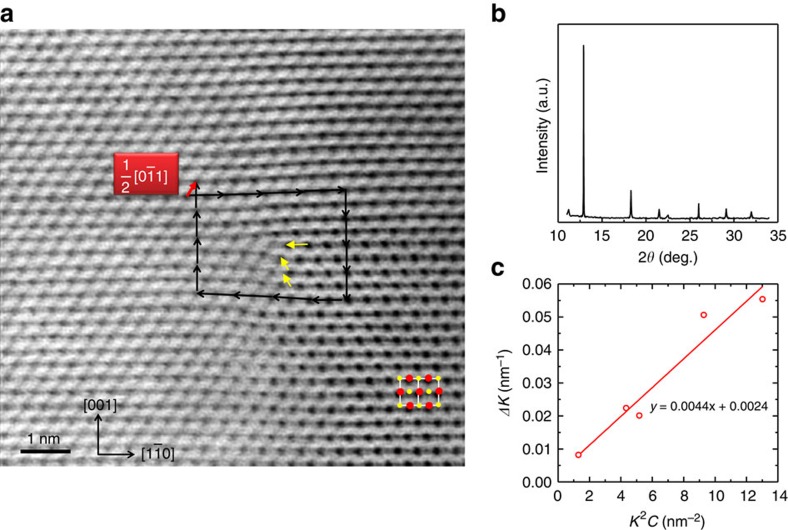
The feature of the dislocation and synchrotron diffraction results for Pb_0.95_Sb_0.033_Se solid solution. A high-resolution annular bright field (ABF) STEM image (**a**) shows the detailed dislocations in the sample with *x*=0.05. The black arrows give a complete Burgers loop of a dislocation and the yellow arrows show one (001) and two (111) extra half planes of atoms. The estimated Burger's vector (red arrow) for the observed dislocations is **B**_**D**_=1/2[0
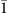
1]. The inset shows the structural model of PbSe projected in [110] direction with red dots standing for Pb, whereas yellow ones for Se. Neither precipitates nor nanometre-scale grains are observed. The synchrotron XRD pattern (**b**) and the peak broadening analysis by the modified Williamson–Hall plot (red line) in which the slope and intercept reveal the dislocation density and the crystallite size (**c**).

An example of a typical dislocation in the high-resolution annular bright field scanninge TEM (STEM) image in Fig. 2a. Starting from the core (centre of the image) of the dislocation, one (001) and two (111) extra half planes of atoms are observed and marked by yellow arrows. The Burgers vectors of the dislocation is determined to be **B**_**D**_=1/2[0
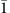
1], which is generally observed in NaCl structures^47^. Based on a few low magnification STEM images, the average areal dislocation density (*N*_D_) is estimated to be ∼4 × 10^12^ cm^−2^; this is an extremely high value compared with that typically reported in semiconductors^30^.

To estimate the density of dislocations more macroscopically and to compare with that determined by the local TEM observations, synchrotron XRD (Fig. 2b) was carried out and the modified Williamson–Hall model^48^,^49^ was employed for the high *zT* sample Pb_0.95_Sb_0.033_Se. According to this method, the broadening of the diffraction peaks strongly relate to the dislocation density and the crystallite size via: *ΔK*=0.9/*d*+(*πA*^2^*B*_D_^2^/2)*N*_D_^1/2^*K*^2^*C*±*O*(*K*^4^*C*^2^) with details shown in Supplementary Table 3. With the experimentally measured *K* and *ΔK*, the dislocation density can be determined via the slope of the modified Williamson–Hall plot as shown in Fig. 2c. The estimated dislocation density is ∼5 × 10^12^ m^−2^ for Pb_0.95_Sb_0.033_Se, which agrees well with the result determined by the TEM observations. Similarly, the modified Williamson–Hall plots, according to our normal XRD results, qualitatively show that the density of dislocations for Pb_1−*x*_Sb_2*x*/3_Se (*x*=0∼0.07) solid solutions increase with increasing Sb concentration (Supplementary Fig. 2).

### Lattice thermal conductivity and modelling

The lattice thermal conductivity (Fig. 1c), determined by subtracting the electronic contribution via the Wiedemann–Franz law from the measured thermal conductivity, decreases with increasing Sb_2_Se_3_ content, meaning a strengthened phonon scattering with increasing *x*. To ensure that the uniformly distributed dense dislocations are indeed responsible for the observed ultralow lattice thermal conductivity, modelling is carried out based on the Debye approximation with different scattering sources for phonons.

First, the literature values of *κ*_L_, which are an average on a few degenerately doped polycrystalline PbSe samples, is used to estimate the ratio (=4) of N- to U-processes for the phonon–phonon scattering^36^. Second, the point defect scattering parameters including both mass and strain contributions are determined according to the literature^50^,^51^,^52^. Third, the scattering by dislocations can be calculated^21^ with the experimental Burger vector and the dislocation density. Klemens' expression for edge dislocation scattering was used. Besides our measured transverse and longitudinal sound velocities of *υ*_L_=3,150 ms^−1^ and *υ*_T_=1,600 ms^−1^, respectively, other parameters including Gruneisen parameter of 1.7 (ref. 53) and Poisson ratio of 0.243(ref. 54) are taken from the literature. More details for the modelling can be found in the Supplementary Tables 1 and 2.

Next, one can calculate the temperature-dependent lattice thermal conductivity using the following equation^55^:


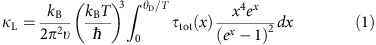


where *k*_B_ is the Boltzmann constant, *υ* is average sound speed, *ℏ* is the reduced Plank constant, *θ*_D_ is Debye temperature, *τ*_tot_ is the total relaxation time and *x*=*ℏ*ω/*k*_B_*T*. The total relaxation time includes the contributions from phonon–phonon, point defect and dislocation scattering by:


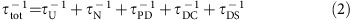


**Figure 3:**
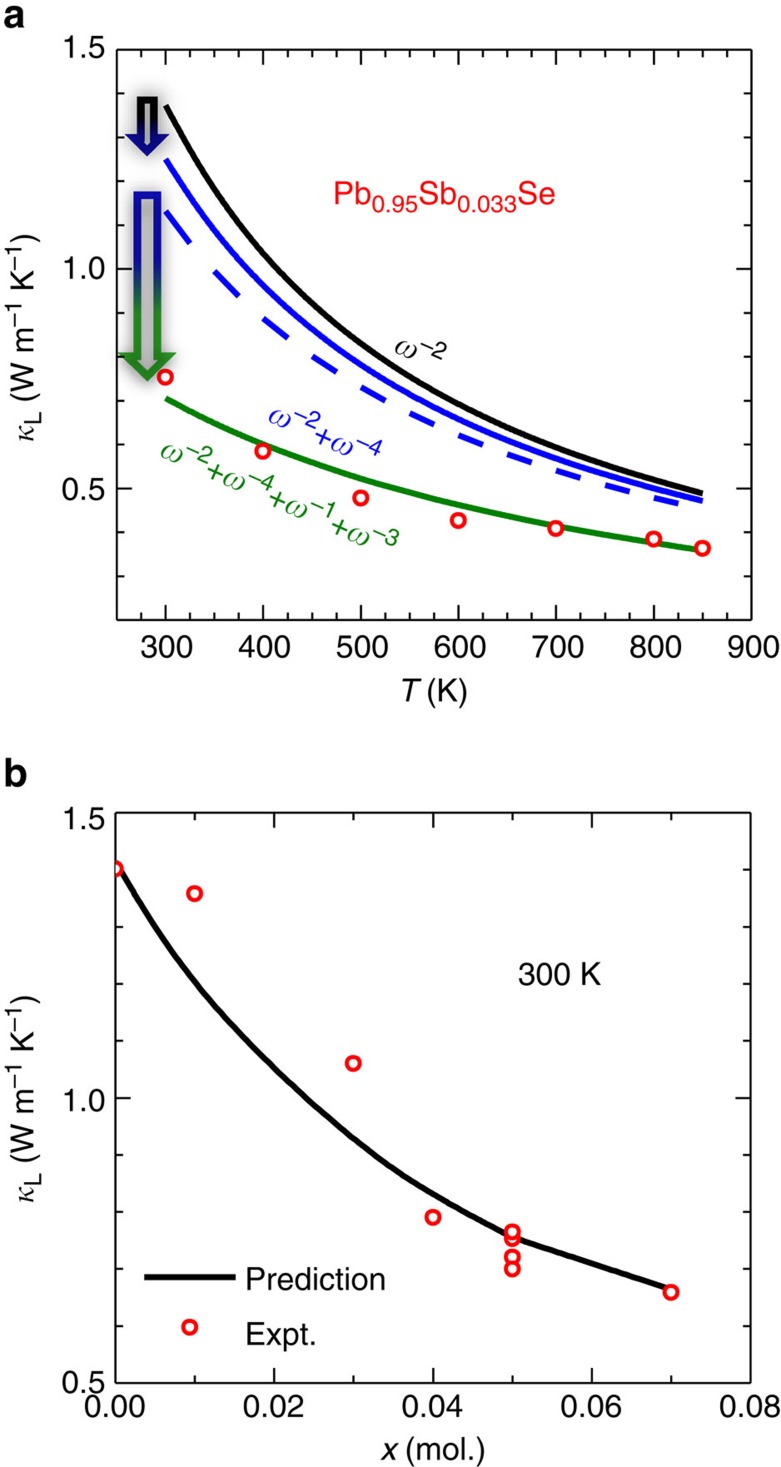
Lattice thermal conductivity and model prediction. Temperature- (**a**) and composition- (**b**) dependent lattice thermal conductivity (symbols) for Pb_1−*x*_Sb_2*x*/3_Se solid solution. Taking into account the frequency (*ω*)-dependent terms for phonon relaxation time including *ω*^−2^ for phonon–phonon (black line in Fig. 3a), *ω*^−4^ for point defect (solid blue line) and *ω*^−1^+*ω*^−3^ for dislocation scattering (olive line), a model based on the Debye approximation (curves) predicts the experimental results. The dashed blue line shows the model prediction, assuming all the Pb vacancies stabilize as random point defects rather than dislocations. The comparison of the dislocations between the *x*=0.05 (Fig. 1a) and the *x*=0.03 (Fig. 1b) samples indicates a nearly linear increase in dislocation density with increasing *x*, further enabling a reliable model prediction on *κ*_L_ versus *x*.

As shown in Fig. 3a, the model prediction agrees well with the measurements for the high thermoelectric figure of merit sample (*x*=0.05). It is then clear that the lattice thermal conductivity (*κ*_L_) additionally decreases by at least 30% in the entire temperature range due to dislocation scattering. Assuming all the initially produced vacancies remains in the solid solution as random point defects, the lattice thermal conductivity is predicted to be as the blue dashed line (Fig. 3a), which is well above the model prediction including the contribution from dislocation scattering (green solid line). Therefore, dislocation scattering is believed to be the main reason for the observed *κ*_L_ reduction. Importantly, the nearly full phonon frequency spectrum scattering leads to a comparable *κ*_L_ with the theoretical minimum (*κ*_L_^min^=0.37 Wm^−1^ K^−1^) obtained by the Cahill's model^37^. Comparing the dislocations in samples with *x*=0.03 (Fig. 1b), *x*=0.04 (Supplementary Fig. 3a,b), *x*=0.05 (Fig. 1a) and *x*=0.07 (Supplementary Fig. 3c,d), a roughly linear increase in the density of the dislocations (*N*_D_) with increasing *x* is observed. Using this simple linear approximation between *N*_D_ and *x*, the above model further enables a nice prediction on *κ*_L_ versus *x* at a given temperature, as shown in Fig. 3b. This confirms the dislocation scattering is indeed helpful for reducing the lattice thermal conductivity.

The model-predicted frequency-dependent accumulative reduction in *κ*_L_ (Supplementary Fig. 4) indeed shows the significant effect of dislocations for mid-frequency phonon scattering in Pb_0.95_Sb_0.33_Se. The predicted accumulative *κ*_L_ due to Umklapp and normal scattering in pure PbSe, further helps us understand the important range of mean free path^56^ that contributes to heat conduction. It is also worthy to note that the mechanical strength does not degrade a lot due to the existence of dense in-grain dislocations here (Supplementary Table 4).

### Transport properties

**Figure 4:**
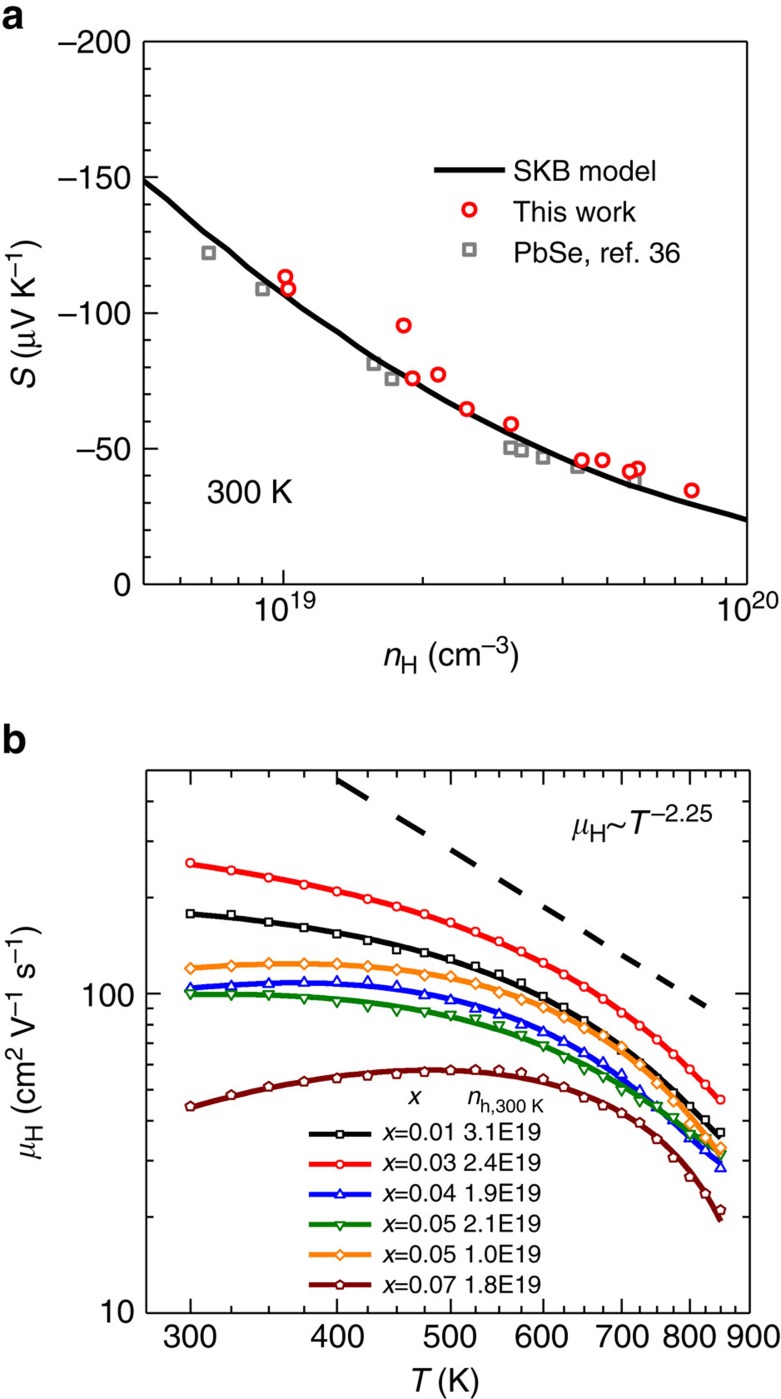
Electrical properties for Pb_1−*x*_Sb_2*x*/3_Se. Hall carrier concentration-dependent Seebeck coefficient (**a**) at room temperature and temperature-dependent Hall mobility (**b**) for Pb_1−*x*_Sb_2*x*/3_Se solid solution. Although the band structure of PbSe remains nearly immune to alloying with Sb_2_Se_3_, the resulting dislocation scattering reduces the mobility.

In spite of the strong scattering of phonons, one needs also to be careful when evaluating the thermoelectric properties, because the formation of solid solutions may change the band structure, whereas the existence of dislocations may affect the scattering of charge carriers. To clarify this possibility, the Hall carrier concentration-dependent room-temperature Seebeck coefficient is shown in Fig. 4a. It is seen that both the literature PbSe^36^ and the current Pb_1−*x*_Sb_2*x*/3_Se solid solution show nearly the same relationship between Seebeck coefficient and Hall carrier concentration. This indicates the band parameters such as the density-of-states effective mass remains unchanged. Therefore, they both can be described by a single Kane band model.

The temperature-dependent Hall mobility (Fig. 4b) helps understand the carrier scattering mechanism. With increasing *x*, thus an increase in dislocation density, it is shown that at *T*<500 K the Hall mobility (*μ*_H_) decreases much slower with increasing temperature as compared with a relationship of *μ*_H_∼*T*^−2.25^ that is normally expected for *n*-PbSe^53^. This can be understood, because a temperature-dependent mobility of *μ*_H_∼*T*^1.5^ will be observed in case of carrier scattering dominated by dislocations^57^, which is similar with that of ionized impurity scattering. This type of scattering can be effectively screened here, because of the high dielectric constant of PbSe^53^; therefore, a high mobility can still be obtained. Similar dielectric screening effects for a high mobility has been found in semiconductors and graphene^58^,^59^,^60^. However, the temperature-dependent Hall mobility in Pb_1−*x*_Sb_*x*_Se^40^, without dense dislocations as in Pb_1−*x*_Sb_2*x*/3_Se, does not indicate a *T*^1.5^-type contribution to the Hall mobility. The electronic transport of Pb_1−*x*_Sb_*x*_Se^40^ without dislocations show normal *n*-type doped PbSe^36^ behaviour.

**Figure 5:**
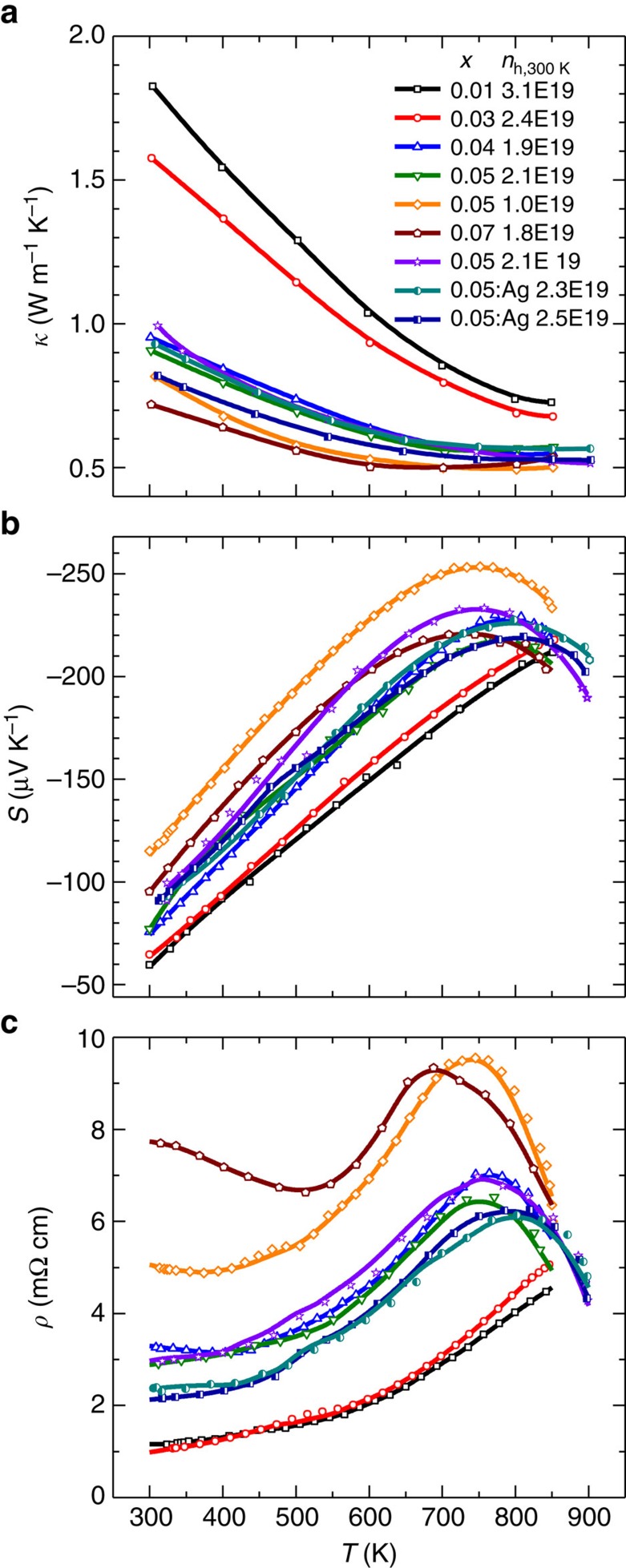
Transport properties as a function of temperature. Temperature-dependent thermal conductivity (**a**), Seebeck coefficient (**b**) and resistivity (**c**) for Pb_1−*x*_Sb_2*x*/3_Se solid solutions.

The temperature-dependent thermal conductivity (*κ*), Seebeck coefficient (*S*) and resistivity (*ρ*) are shown in Fig. 5a–c, respectively. With increasing *x*, the reduced thermal conductivity is largely from the lattice thermal conductivity reduction due to the vacancy-induced dislocations. It should be noted that the Lorenz factor is determined by the single Kane band model to estimate the electronic thermal conductivity (*κ*_E_). At *T*<600 K, the decrease in resistivity in the heavily alloyed samples can be explained by the resulting dislocation scattering on carriers (Fig. 4b). The decrease in *ρ* and *S* at *T*>700 K is probably due to the existence of minority carriers, which is normally seen in narrow band gap semiconductors.

## Discussion

In summary, this work presents a method to create uniformly distributed dense dislocations throughout the entire grains of Pb_1−*x*_Sb_2*x*/3_Se solid solutions by the introduction of cation vacancies. Owing to the strong phonon scattering by dislocation cores and strain fields, the lattice thermal conductivity reaches the theoretical minimum and, therefore, a high figure of merit *zT* is obtained. Unlike previously demonstrated approaches focusing on the scattering of phonons with either low or high frequencies, this work proposes a vacancy engineering strategy for well-dispersed dense dislocations, enabling an effective demonstration of mid-range frequency phonon scattering for low lattice thermal conductivity. This strategy emphasizes a thermal effect, which is independent of other existing strategies, such as band engineering, for increasing the electrical performance^2^. Therefore, a combination with other strategies offers great potential for further improvements of thermoelectrics.

## Methods

### Synthesis

Starting with pure elements (Pb 99.99%, Sb 99.99%, Se 99.999%), Pb_1−*x*_Sb_2*x*/3_Se solid solutions with *x* from 0 to 9% were prepared by melting at 1,400 K for 6 h, followed by quenching in water and annealing at 1,023 K for 2 days. An Ag doping of ≤1% at the Pb site was also used to help control the carrier concentration precisely. The ingot materials were ground into fine powders for hot pressing^58^ at 973 K for 1 h under a uniaxial pressure of ∼90 MPa under vacuum. The disk samples for measurements were ∼2 mm in diameter and ∼1.5 mm in thickness, and the density (*d*) is higher than 96% of theoretical value.

### Characterization

The phase impurity was characterized by lab XRD (Dandong Haoyuan Instrument Co. Ltd). The samples for (S)TEM observation were prepared by mechanical polishing, dimpling and ion milling with liquid nitrogen. TEM and STEM imaging including in high-angle annular dark field and annular bright field modes, as well as energy dispersive X-ray spectroscopy element mapping were carried out by JEOL ARM 200 equipped with a probe corrector and an image corrector. Synchrotron XRD measurements were carried out at the beamline number 14B of the Shanghai Synchrotron Radiation Facility. The energy of monochromatic X-ray beam was 10 keV, corresponding to a wavelength of 0.687 nm. The reflection mode was applied and the signal was recorder by a NaI(Tl) scintillation detector. The beam size is 0.5 × 1 mm. The line profiles were recorded with a step size of 0.02° and a dwell time of 3 s.

### Transport property measurements

The electrical properties including Seebeck coefficient, resistivity and Hall coefficient were simultaneously measured on the samples during both heating and cooling in vacuum. The Seebeck coefficient in the in-plane direction was obtained from the slope of the thermoelectric voltage versus temperature gradients^59^. The in-plane resistivity and Hall coefficient were measured using the Van der Pauw technique under a reversible magnetic field of 1.5 T. The uncertainty of each transport property measurement is ∼5%. The thermal diffusivity *D*_T_ was measured by the laser flash method (Netzsch LFA 457) and the thermal conductivity *κ* was calculated from *κ*=*dD*_T_*C*_p_, where *C*_p_ is the heat capacity determined by *C*_p_(*k*_B_/atom)=(3.07+0.00047(*T*/K-300))^60^,^61^. The sound velocity was measured by an ultrasonic pulse-receiver (Olympus-NDT) equipped with an oscilloscope (Keysight). The uncertainty for each measurement of transport property is ∼5%.

### Data availability

The data that support the findings of this study are available from the corresponding author upon request.

## Additional information

**How to cite this article:** Chen, Z. *et al*. Vacancy-induced dislocations within grains for high-performance PbSe thermoelectrics. *Nat. Commun.* **8,** 13828 doi: 10.1038/ncomms13828 (2017).

**Publisher's note:** Springer Nature remains neutral with regard to jurisdictional claims in published maps and institutional affiliations.

## Supplementary Material

Supplementary Information Supplementary Figures, Supplementary Tables, Supplementary Discussion, Supplementary References.
