# Supplementary material for: Vacancy-induced dislocations within grains for high-performance PbSe thermoelectrics
Source: Nat Commun. 2017 Jan 4;8:13828. doi: 10.1038/ncomms13828 (PMC5216132; doi:10.1038/ncomms13828)
Supplement: Supplementary Information — Supplementary Figures, Supplementary Tables, Supplementary Discussion, Supplementary References. [file ncomms13828-s1.pdf]

Supplementary Figures

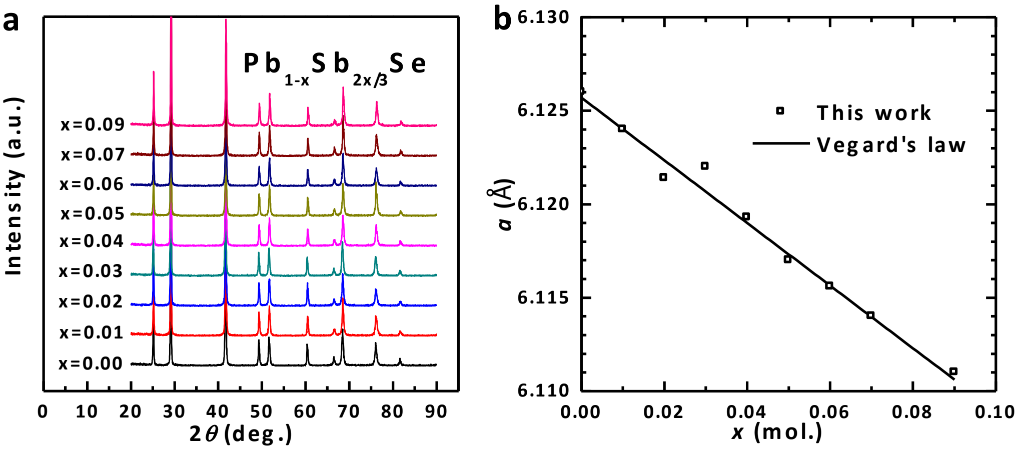

Supplementary Figure 1. Phase characterization. The X-ray diffraction patterns (a) and lattice parameters for $Pb_{1-x}Sb_{2x/3}Se$ (x = 0~0.09) solid solutions.

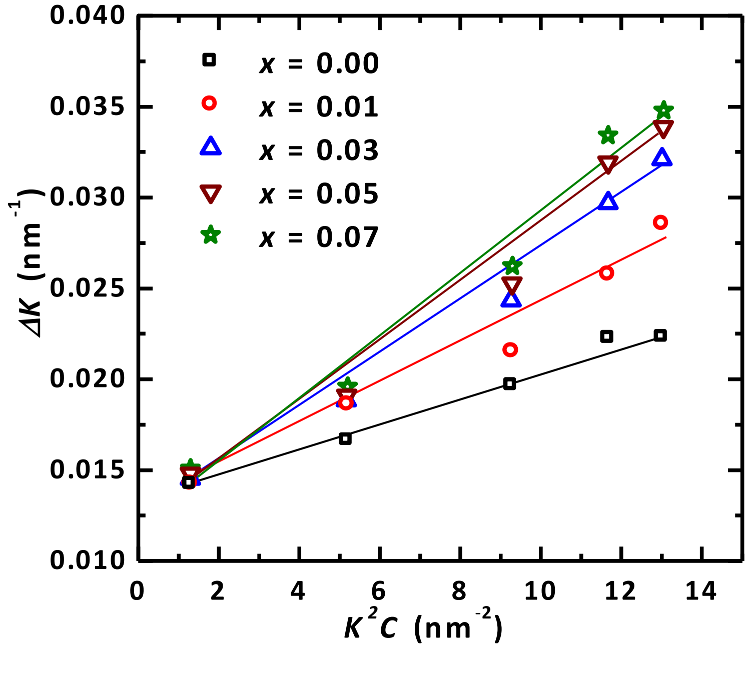

Supplementary Figure 2. The modified Williamson-Hall plots. The peak broadening analysis by the modified Williamson-Hall plots for $Pb_{1-x}Sb_{2x/3}Se$ (x = 0~0.07) solid solutions according to the XRD data.

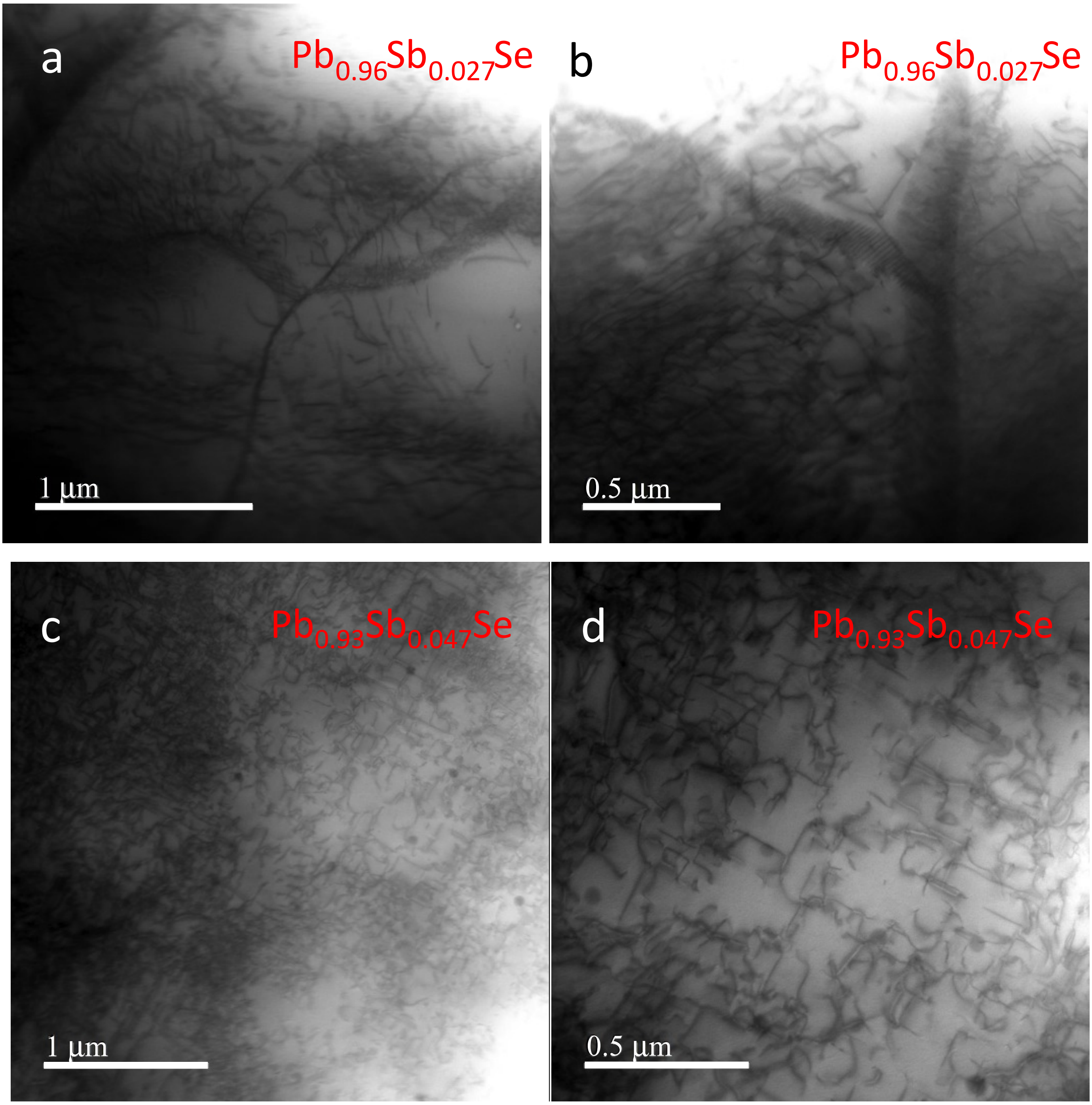

Supplementary Figure 3. Microstructures of $Pb_{0.96}Sb_{0.027}Se$ and $Pb_{0.93}Sb_{0.047}Se$. Dislocations in $Pb_{0.96}Sb_{0.027}Se$ (a, b) and $Pb_{0.93}Sb_{0.047}Se$ (c, d), confirming the increased dislocation density with increasing Sb_2Se_3 concentration.

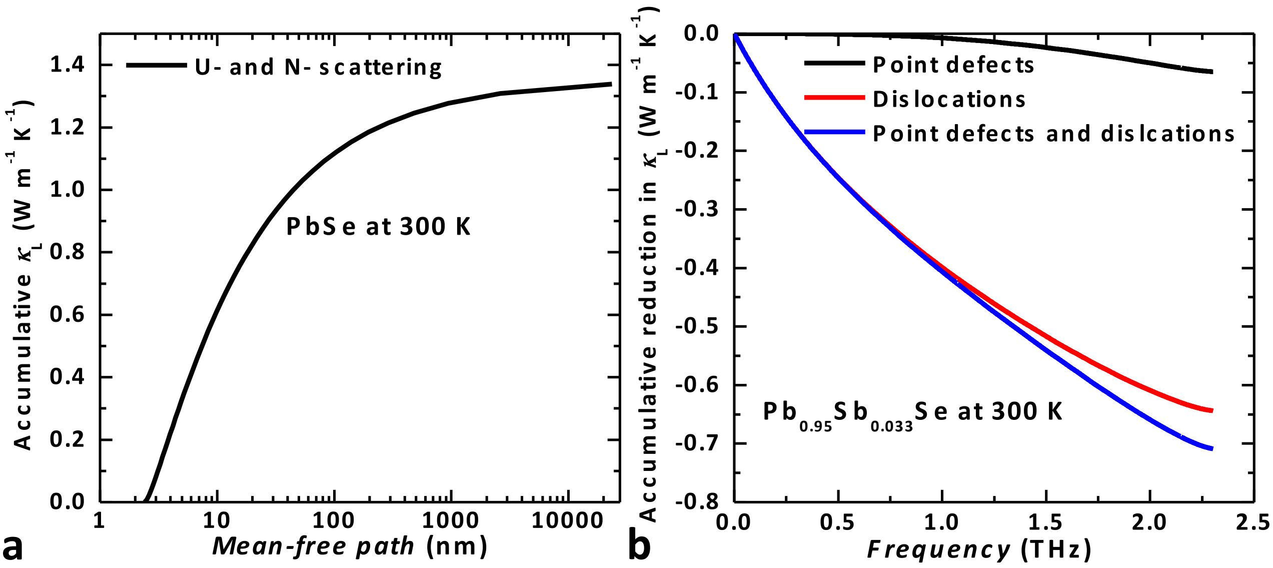

Supplementary Figure 4. Predictions of lattice thermal conductivity using Born-von Karman approximation. Predicted mean-free path dependent accumulative lattice thermal conductivity for PbSe (a), and the predicted frequency dependent accumulative reduction in the lattice thermal conductivity for $Pb_{0.95}Sb_{0.33}Se$ due to point defects and/or dislocations (b). The modeling is based on a Born-von Karman approximation and the predictions are for 300 K.

Supplementary Tables

Supplementary Table 1. Equations for phonon relaxation times (τ) associated with different types of scattering processes, where τ_{U}, τ_{N}, τ_{PD}, τ_{DC} and τ_{DS} are the relaxation times due to the scattering of Umklapp processes, Normal processes, point defects, dislocation cores and dislocation strains, respectively.

Types of scattering mechanisms	Relaxation times ($\tau(s^{-1})$)
Umklapp processes	$\tau_{U}{}^{-1} = \frac{2}{(6\pi^2)^{1/3}} \frac{k_B \bar{V}^{1/3} \gamma^2 \omega^2 T}{\bar{M} v^3}$
Normal processes	$\tau_{N}{}^{-1} = \frac{2}{(6\pi^2)^{1/3}} \frac{k_B \bar{V}^{1/3} \gamma^2 \omega^2 T}{\bar{M} v^3}$
Point defects	$\tau_{PD}{}^{-1} = \frac{\bar{V}\omega^4}{4\pi v^3} \sum_i x_i \left[\left(\frac{M_i - M}{M} \right)^2 + \varepsilon` \left(\frac{a_i - a}{a} \right)^2 \right]$
Dislocation cores	$\tau_{DC}{}^{-1} = N_D \frac{\bar{V}^{4/3}}{v^3} \omega^3$
Dislocation strains	$\tau_{DS}{}^{-1} = A \times B_D{}^2 \gamma^2 \omega \left[\frac{1}{2} + \frac{1}{24} \left(\frac{1-2r}{1-r} \right)^2 \left(1 + \sqrt{2} \left(\frac{v_L}{v_T} \right)^2 \right)^2 \right]$

Supplementary Table 2. Parameters used for the Debye modeling.

Parameters	Description	Values	Ref.
β	ratio of N- to U- processes	4	[1], estimated
$\bar{V}$	Average atomic volume of $Pb_{1-x}Sb_{2x/3}Se$	$a_i^3/8$ m^3	-
$\bar{M}$	Average atomic mass for $Pb_{1-x}Sb_{2x/3}Se$	$M_{Pb1-xSb2x/3Se}/(2\times6.023\times10^{23})$ kg	-
v	Average sound speed	1787 m s^{-1}	This work
v_L	Longitudinal sound speed	3150 m s^{-1}	This work
v_T	Transverse sound speed	1600 m s^{-1}	This work
γ	Gruneisen parameter	1.7	[2]
x_i	Impurities concentration in solid solutions	$x_{Sb} \leq 0.07$	This work
M_i	Atomic mass of impurities	M_{Sb} = 121.67 g mol^{-1}	-
M	Atomic mass of matrix	M_{Pb} = 207.2 g mol^{-1}	-
$\varepsilon`$	Anharmonic parameter	64	[3]
a_i	Lattice parameters for $Pb_{1-x}Sb_{2x/3}Se$	6.1257-0.1675x_i Å	This work
A	Lattice parameters for PbSe	6.126 Å	This work
N_D	Dislocation density of $Pb_{1-x}Sb_{2x/3}Se$	$[60(x_i-0.01)+1]\times10^{12}$ $x_i \geq 0.01$ cm^{-2}	This work
B_D	Burgers vector	4.33×10^{-10} m	This work
A	Pre-factor for dislocation scattering	0.96	[4]
r	Poisson's ratio	0.243	[2]

Supplementary Table 3. Parameters used for the modified Williamson-Hall model.

Parameters	Description	Value					Ref.
θ_B	Diffraction angle at the exact Bragg position	6.45°	9.14°	12.99°	14.56°	15.98°	This work
hkl	Indices of crystal plane	(200)	(220)	(400)	(420)	(422)	-
$\Delta 2\theta$	Full width at half-maximum (FWHM) of the corresponding diffraction peak at θ_B	0.032°	0.089°	0.081°	0.206°	0.227°	This work
λ	Wavelength of the synchrotron X-ray	6.87 Å					-
K	$K=2\sin\theta_B/\lambda$	$2\sin\theta_B/\lambda$					[5, 6]
ΔK	$\Delta K=(\Delta 2\theta)\cos\theta_B/\lambda$	$(\Delta 2\theta)\cos\theta_B/\lambda$					[5, 6]
A	Parameter determined by the effective outer cut-off radius of dislocations	2.6					[7]
B_D	Burgers vector	4.33×10^{-10} m					This work
C	Average dislocation contrast factor	$C_{h00}(1-q(h^2k^2+h^2l^2+k^2l^2)/(h^2+k^2+l^2)^2)$					[5, 6]
C_{h00}	Average dislocation contrast factor corresponding to the *h00* reflection determining by elastic modulus	0.12148					This work, [5, 6]
q	Parameter determined by the elastic modulus	-2.7					This work, [5, 6]
c_{11}		123.7 GPa					
c_{12}	Elastic modulus	19.3 GPa					[8]
c_{44}		15.9 GPa					
O	Non-interpreted higher-order error terms	Not included in this work					[5, 6]
d	Average crystallite size	375.00 nm					This work, fitted
N_D	Dislocation density	5.0×10^{12} m^{-2}					This work, fitted

Supplementary Table 4. Mechanical strength measured by modified small punch (MSP) technique for several materials at room temperature.

Composition of compounds	Thickness of samples (mm)	Load at failure (N)	MSP strength (MPa)
PbSe	0.935	32.8	27.8575
$Na_{0.02}Pb_{0.98}Te$	1.04	32.8	22.5164
$Pb_{0.98}Sb_{0.013}Se$	0.725	16.8	23.7314
$Pb_{0.95}Sb_{0.033}Se$	0.99	26.5	20.0755
$Pb_{0.95}Sb_{0.033}Se$	0.97	23.6	18.5445
$Pb_{0.93}Sb_{0.047}Se$	0.945	22.8	18.9567
$Pb_{0.93}Sb_{0.047}Se$	0945	19.8	16.4624

Supplementary Discussion

To better understand the mean free path and the frequency dependent lattice thermal conductivity (κ_L) accumulation, it is believed to be more precise if taking the effect of reduced phonon group velocity at high phonon energies into account [9, 10]. This leads the Born-von Karman [11] dispersion relationship to be more reliable than that of Debye model. This improvement has been adopted to understand the lattice thermal conductivity of bulk and low-dimensional thermoelectrics or metals including Si-Ge [12] and PbTe [13], silver[14] and Al-Si etc.[15]. Using the same method, we modeled the phonon transport for PbSe, based on a Born-von-Karman type phonon dispersion of $\omega = 2vq/\pi Sin(2q/q_c\pi)$ rather than the Debye type assuming $\omega = vq$, where ω is the phonon frequency, v is the sound velocity, q is the wave vector and q_c is the cut-off wave vector. The predicted accumulative κ_L due to Umklapp and Normal scattering in pure PbSe, helps us understand the important range of mean free path that contributes to heat conduction. Furthermore, the predicted phonons frequency dependent accumulative reduction in κ_L distinguishes the effect of each scattering mechanism.

As shown in the Supplementary Fig. 4a, 50% of the heat in pure PbSe is carried by phonon of mean-free path up to 13 nm and the central 80% heat is carried by mean-free path (MFP) between 4 nm and 400 nm at 300 K. As compared with the available prediction by first-principles calculations[16], which includes the contributions of optical phonons (with even shorter MFPs) and results in a higher lattice thermal conductivity, the current model prediction shows a very good agreement on the normalized κ_L-accumulation within the overlapped range of MFP. The randomly distributed dense in-grain dislocations here roughly enable a range of mean-free path to be achieved for reducing the lattice thermal conductivity by 50% at the 300 K (Fig. 3a). It is shown that dense in-grain dislocations, indeed lead to an effective scattering of phonons with mid-frequencies and therefore a significantly reduced lattice thermal conductivity (Supplementary Fig. 4b).

The mechanical property measurements were carried out by modified small punch (MSP) technique [17], a method has been successfully used to characterize the mechanical strength of thermoelectric materials [18, 19]. For the MSP measurements, the disk sample was supported by a die with a center hole of 3.93 mm in diameter, and was punched by a cylindrical pressure head of 2.35 mm in diameter with a speed of 0.05 mm min^{-1}. All of the specimens were fine polished and the load was monitored by a high-accuracy transducer. The MSP strength σ_{MSP} can be calculated via: $\sigma_{MSP} = 3P_{max}/(2\pi t^2)[1-(1-v^2)/4\times b^2/a^2+(1+v)\ln(a/b)]$, where P_{max} is the measured load at failure, t is the thickness of the sample, v is the Poisson's ratio which is estimated as 0.243[2] for PbSe-based materials and as 0.218[2] for PbTe-based materials, a is radius of the center hole and b is the radius of pressure head, respectively.

The mechanical strength is obtained by averaging 3~5 samples for each composition, and the results for $Pb_{1-x}Sb_{2x/3}Se$, PbSe and PbTe are shown in Supplementary Table 4. One may claim that the mechanical strength of $Pb_{1-x}Sb_{2x/3}Se$ decreases a little with increasing density of dislocations (increasing x), however, the strength for samples with dense dislocations is still comparable to that of PbTe without dislocations. Therefore, dense in-grain dislocations here do not degrade the mechanical strength to be unacceptable.

Supplementary References

1. Wang H, Pei Y, LaLonde AD, Snyder GJ. Weak electron-phonon coupling contributing to high thermoelectric performance in n-type PbSe. *Proc Natl Acad Sci USA* **109**, 9705-9709 (2012).
2. Ravich YI, Efimova BA, Smirnov IA. *Semiconducting Lead Chalcogenides* (Plenum Press, 1970).
3. Wang H, Wang J, Cao X, Snyder GJ. Thermoelectric alloys between PbSe and PbS with effective thermal conductivity reduction and high figure of merit. *Journal of Materials Chemistry A* **2**, 3169 (2014).
4. Kemp WRG, Klemens PG, Tainsh RJ. The lattice thermal conductivity of copper alloys: Effect of plastic deformation and annealing. *Philos Mag* **4**, 845-857 (1959).
5. Ungár T, Dragomir I, Revesz A, Borbély A. The contrast factors of dislocations in cubic crystals: the dislocation model of strain anisotropy in practice. *Journal of applied crystallography* **32**, 992-1002 (1999).
6. Ungar T, Ott S, Sanders P, Borbély A, Weertman J. Dislocations, grain size and planar faults in nanostructured copper determined by high resolution X-ray diffraction and a new procedure of peak profile analysis. *Acta Mater* **46**, 3693-3699 (1998).
7. Ribárik G. Modeling of diffraction patterns based on microstructural properties. (ed^(eds). Institute of Physics (2008).
8. Lippmann G, Kästner P, Wanninger W. Elastic constants of PbSe. *physica status solidi (a)* **6**, K159-K161 (1971).
9. Ashcroft N, Mermin N. *Solid State Physics* (Harcourt Brace College, 1976).
10. Ziman JM. *Electrons and phonons: the theory of transport phenomena in solids* (Oxford University Press, 1960).
11. Born M, von Kármán T. On fluctuations in spatial grids. *Physikalische Zeitschrift* **13**, 18 (1912).
12. Dames C. Theoretical phonon thermal conductivity of Si/Ge superlattice nanowires. *J Appl Phys* **95**, 682 (2004).
13. Greig D. Thermoelectricity and Thermal Conductivity in the Lead Sulfide Group of Semiconductors. *Phys Rev* **120**, 358-365 (1960).
14. Leighton RB. The Vibrational Spectrum and Specific Heat of a Face-Centered Cubic Crystal. *Rev Mod Phys* **20**, 165-174 (1948).
15. Reddy P, Castelino K, Majumdar A. Diffuse mismatch model of thermal boundary conductance using exact phonon dispersion. *Appl Phys Lett* **87**, 211908 (2005).
16. Tian Z, Garg J, Esfarjani K, Shiga T, Shiomi J, Chen G. Phonon conduction in PbSe, PbTe, and $PbTe_{1-x}Se_x$ from first-principles calculations. *Phys Rev B* **85**, (2012).
17. Li J-F, Pan W, Sato F, Watanabe R. Mechanical properties of polycrystalline Ti_3SiC_2 at ambient and elevated temperatures. *Acta Mater* **49**, 937-945 (2001).
18. Pan Y, Wei T-R, Cao Q, Li J-F. Mechanically enhanced p- and n-type Bi_2Te_3-based thermoelectric materials reprocessed from commercial ingots by ball milling and spark plasma sintering. *Materials Science and Engineering: B* **197**, 75-81 (2015).
19. Li J*, et al.* BiSbTe‐Based Nanocomposites with High ZT: The Effect of SiC Nanodispersion on Thermoelectric Properties. *Adv Funct Mater* **23**, 4317-4323 (2013).
